# Supplementary material for: Reflections Based on Pollution Changes Brought by COVID-19 Lockdown in Shanghai
Source: Int J Environ Res Public Health. 2021 Oct 10;18(20):10613. doi: 10.3390/ijerph182010613 (PMC8536036; doi:10.3390/ijerph182010613)

Supllimentary materials

SFig.1 NMDS analysis of different polutants before and during lockdown period

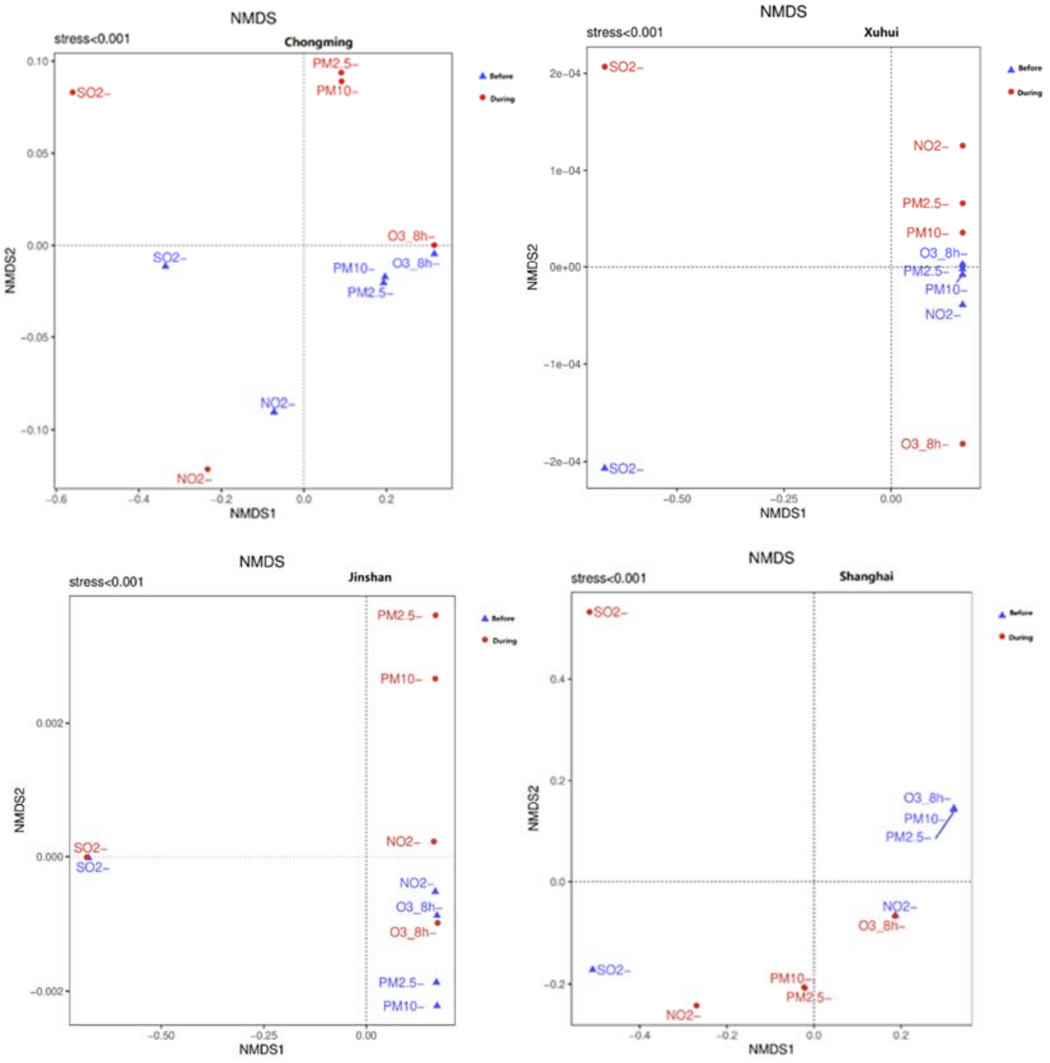

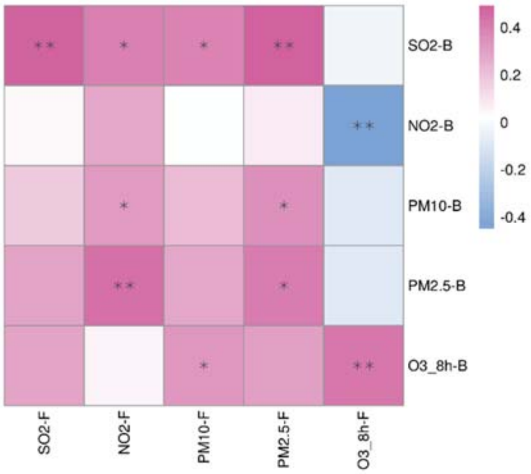

**
*
*
**
SO2-B
**
NO2-B
*
*
PM10-B
**
*
PM2.5-B
*
**
O3_8h-B
SO2-F
NO2-F
PM10-F
PM2.5-F
O3_8h-F
0.4
0.2
0
-0.2
-0.4

SFig 2 . Clustering analysis of different pollutants before (F) and during lockdown (B)

in differernt functional parts of Shanghai

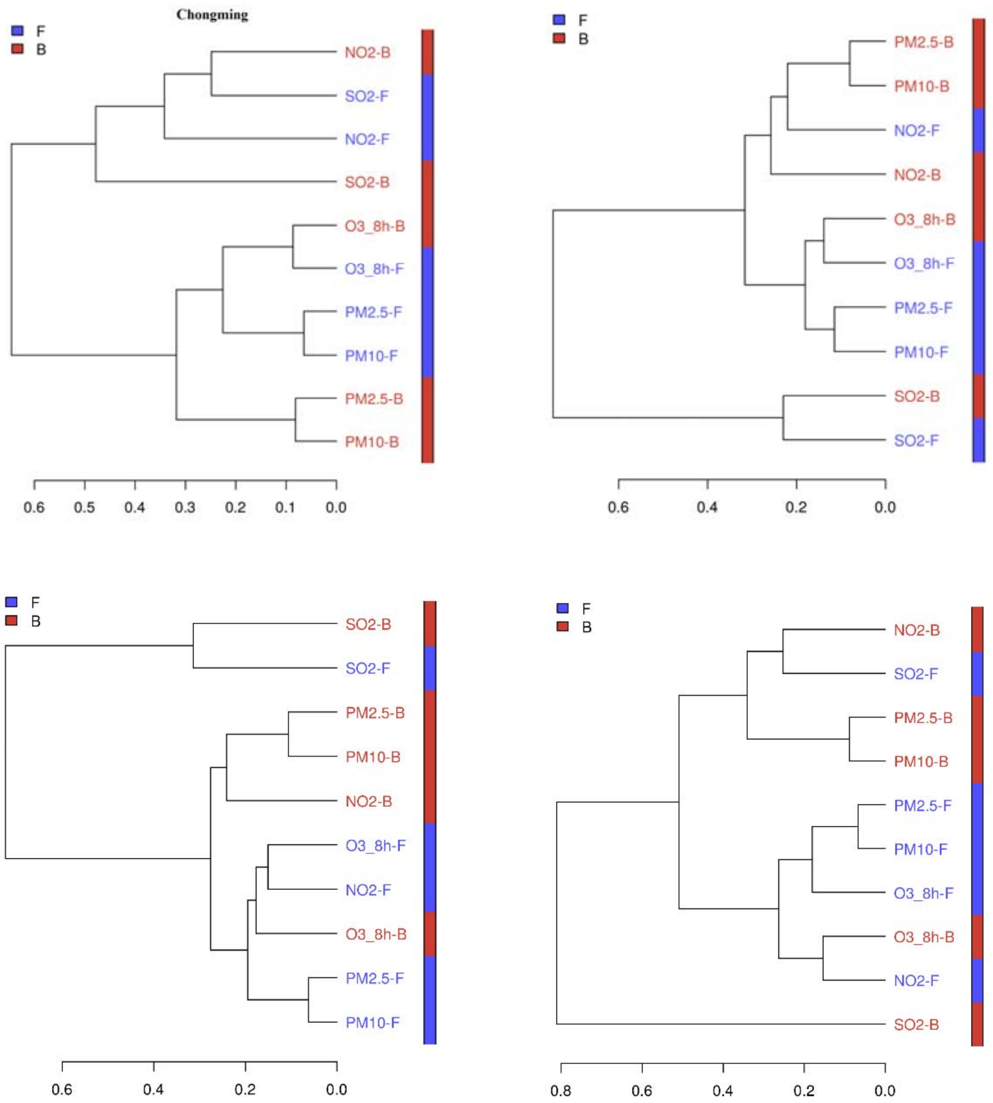

Supplement: Supplementary file 1 [file ijerph-18-10613-s001.zip › ijerph-1352860-supplementary.pdf]
